# Role of Na-Montmorillonite on Microbially Induced Calcium Carbonate Precipitation

**DOI:** 10.3390/molecules26206211

**Published:** 2021-10-14

**Authors:** Guowang Tang, Cangqin Jia, Guihe Wang, Peizhi Yu, Haonan Zhang

**Affiliations:** 1 School of Engineering and Technology, China University of Geosciences, Beijing 100083, China; 3002180015@cugb.edu.cn (G.T.); wanggh@cugb.edu.cn (G.W.); 2011010003@cugb.edu.cn (P.Y.); 3002190015@cugb.edu.cn (H.Z.); 2 Key Laboratory of Deep Geodrilling Technology, Ministry of Land and Resources, China University of Geosciences, Beijing 100083, China

**Keywords:** calcium carbonate, morphology of precipitates, Na-montmorillonite, hydraulic conductivity, bio-mineralization

## Abstract

The use of additives has generated significant attention due to their extensive application in the microbially induced calcium carbonate precipitation (MICP) process. This study aims to discuss the effects of Na-montmorillonite (Na-MMT) on CaCO_3_ crystallization and sandy soil consolidation through the MICP process. Compared with the traditional MICP method, a larger amount of CaCO_3_ precipitate was obtained. Moreover, the reaction of Ca^2+^ ions was accelerated, and bacteria were absorbed by a small amount of Na-MMT. Meanwhile, an increase in the total cementing solution (TCS) was not conducive to the previous reaction. This problem was solved by conducting the reaction with Na-MMT. The polymorphs and morphologies of the CaCO_3_ precipitates were tested by using X-ray diffraction and scanning electron microscopy. Further, when Na-MMT was used, the morphology of CaCO_3_ changed from an individual precipitate to agglomerations of the precipitate. Compared to the experiments without Na-MMT in the MICP process, the addition of Na-MMT significantly reduced the hydraulic conductivity (HC) of sandy soil consolidated.

## 1. Introduction

Portland cement (PC) used as a construction material and for soil improvement has contributed to about 5–8% of total carbon dioxide (CO_2_) emissions, which has triggered the search for an alternative binder instead of PC [1]. A decade ago, microbially induced carbonate precipitation (MICP) became a substitute for PC for soil improvement [2,3,4]. While the physical and chemical properties of the soil improved, MICP can also be used as an agricultural fertilizer [4]. Many studies have indicated that MICP used as a PC alternative can enhance soil strength and decrease soil permeability [5]. Unlike PC, MICP technology can be used at an ambient temperature. Therefore, it is cost-effective. Moreover, compared to PC, the construction process of MICP is simpler due to its low viscosity and the smaller amount of bacteria used [6].

MICP technology used for soil improvement is based on urea hydrolysis catalyzed by the urease of bacteria [7,8]. Numerous studies involving the precipitates of calcium carbonate (CaCO_3_) by bacterial urease have been carried out. However, the reaction mechanism of MICP is still undefined because the physiology and genetics involved are difficult to understand [9]. So far, it has been widely considered that the reaction mechanism of MICP can be concluded in line with Equations (1) and (2) [10,11,12], which summarize the biochemical reaction process of the CaCO_3_ precipitates with bacteria serve as nucleation sites.

(1) $$\mathrm{CO}({\mathrm{NH}}_{2}{)}_{2}+H_{2}O+\mathrm{bacterial}\;\mathrm{urease}\;\to \;{\mathrm{CO}}_{3}^{2-}+2{\mathrm{NH}}_{4}^{+}$$

(2) $${\mathrm{CO}}_{3}^{2-}\;+{\mathrm{Ca}}^{2+}\;\to \;{\mathrm{CaCO}}_{3}\;↓$$

To date, the MICP method has been widely studied in many areas. Previous studies have reported that the unconfined compressive strength and the durability of loose sandy soil particles could be achieved via the MICP process to improve their engineering properties [13,14]. Meanwhile, fissures of cement and concrete can also be remediated by this method [15,16]. Moreover, the types of CaCO_3_ precipitates induced by MICP have also been investigated. As reported in the previous works in the literature, the minerals occur as three non-hydrated CaCO_3_ crystals: calcite, aragonite, and vaterite [12,17,18,19,20,21]. In addition to the above studies, wind erosion and fugitive dust generation are effectively controlled through the MICP process [8,22,23,24,25]. Some research on heavy metals immobilized by MICP has also been conducted [26]. For example, the remediation of copper-contaminated soil has been controlled [27,28] as well as the co-precipitation of strontium based on the MICP process [27,28]. 

In the MICP process, the additives are considered to be critically important. At the same time, it has been indicated that various additives, including PVA fibers [29], water-soluble nacre proteins [30], nanoparticles (NPs) [31], and sodium carboxymethyl cellulose (CMC) [32] contribute to MICP technology. Research has shown that the physical properties (e.g., strength and permeability) of sandy soil have been enhanced with PVA fibers. CMC has improved the amount of CaCO_3_ produced. The water-soluble nacre proteins have controlled the CaCO_3_ precipitate rate. NPs on the CaCO_3_ surface can influence CaCO_3_ transport in the soil. Nevertheless, the uneven distribution of PVA fibers limits their large-scale use. Due to the cost factor and potential environmental issues, the water-soluble nacre proteins NPs and CMC used in the MICP process have been limited to the laboratory scale [33].

Therefore, searching for cheap and efficient additives is of great economic value. Na-montmorillonite (Na-MMT) is low cost and is widely used in industry. Applications have included water pollution treatment, drugs, catalysts, drilling materials, and coatings due to the highly specific surface and the high cation exchange capacity [34]. However, few studies in the literature have reported research on Na-MMT in the MICP process and the improvement of sandy soil.

This paper aims to investigate the effect of Na-MMT in the MICP process. The reaction rate of Ca^2+^ ions, the amount of bacteria adsorbed, the amount of CaCO_3_ precipitates, and the morphology of CaCO_3_ precipitates were assessed via the gravimetric method, a UV spectrophotometer, a pH meter, and permeability measurements. The MICP process with the addition of the Na-MMT samples was compared with the traditional MICP process using various analysis techniques. The morphology and microstructure with and without the Na-MMT samples were evaluated with X-ray diffraction (XRD), Fourier transform infrared spectroscopy (FTIR), and scanning electron microscopy (SEM).

## 2. Materials and Methods

### 2.1. Growth Conditions of Bacteria 

The bacteria strain used was the *Sporosarcina pasteurii* (SP) (ATCC11859) to produce CaCO_3_ precipitates. CASO (15 g Casein peptone, 5 g soy peptone and 5 g NaCl) and 20 g urea were mixed into 1 L deionized water to prepare a culture medium. The final pH of approximately 7.3 was achieved by adjusting the culture medium. The culture medium was sterilized at 121 °C for 30 min. SP was cultured aerobically at 28 °C with a rotation rate of 120 rpm for 18 h. SP in the medium was centrifuged with 5434× *g* for 10 min. The centrifuged bacteria were resuspended in deionized water with an optical density at 600 nm (OD_600_) of 1.0 for future use. The amount of bacteria was expressed by measuring the absorbance of the bacterial solution (turbidimetric method). This principle was mainly based on the fact that the bacterial concentration was directly proportional to the turbidity of the bacterial solution. Therefore, it was also directly proportional to the absorbance. In this paper, the absorbance (OD_600_) of an ultraviolet visible spectrophotometer (model uv-1700, Shimadzu Company, Kyoto, Japan) at a wavelength of 600 nm was used to determine the amount of bacteria. The actual amount of bacteria was converted by Equation (3) [35].

Y = 8.59 × 10^7^·Z^1.3627^ (3)

where Z is the OD_600_ value and Y is the bacterial concentration (bacterial mL^−1^). However, it should be noted that this formula can be used for conversion only when OD_600_ is between 0.2 and 0.8. When OD_600_ exceeds this range, it needs to be diluted before conversion. In this paper, the OD_600_ value is usually used to express the bacterial concentrations directly. All chemical reagents were purchased from Sinopharm Chemical Reagent Co, Ltd. (Shanghai, China). All reagents used in this study were of analytical grade and were used without further purification.

### 2.2. Na-MMT and Sandy Soils Used

The sample used was Na-MMT provided by Zhejiang Fenghong New Material Co Ltd, Zhejiang, China. The sandy soil used was from river sand. The particle size distribution curves and the SEM images of Na-MMT and the sandy soil are shown in Figures S1 and S2. Na-MMT was composed of Na-MMT and SiO_2_, as shown in Figure S3A. FTIR analysis data of Na-MMT is shown in Figure S3B. The identification of Na-MMT was made by comparing the spectrum with IR pattern catalogues (The wavelength number of Na-MMT is shown in Table S1). The BET analysis data of Na-MMT are shown in Figure S4. The results show that Na-MMT’s surface area was 19.6012 m^2^/g.

### 2.3. Precipitation and Consolidation Experiments

A total of 55.5 g CaCl_2_ and 30.03 g urea were dissolved into 1 L deionized water to make the reaction solution (0.5 mol/L). The total cementing solution (TCS) of different quantities (TCS = 100, 200, 300, and 400 g) consisted of the mixture of the reaction solution and the bacterial suspension (bacterial suspension (OD_600_ = 1.0): reaction solution (0.5 mol/L) = 1:4). When TCS was prepared, Na-MMT was immediately added to TCS. Afterwards, experiments related to the CaCO_3_ precipitate and bacterial adsorption were carried out at 25 °C in beakers.

To study the effect of the Na-MMT content, various Na-MMT contents of 0%, 0.25%, 0.5%, 0.75%, and 1% were used in 300 g TCS. The amount of bacteria was determined after adding Na-MMT, and the Ca^2+^ ion contents and the pH were tested over time. 

The effect of TCS quantities with and without 0.25% Na-MMT was also studied by changing TCS quantities at 100 g, varying from 100 to 400 g. The amount of bacteria was determined after adding Na-MMT and the Ca^2+^ ion contents were tested over time.

After the experiments (27 h), the CaCO_3_ precipitates were obtained by filtering the solution and then drying it at 105 °C for 24 h. Consolidated samples were achieved by full contact flexible molds [36]. The specification of the mold was a diameter of 30 mm and a height of 60 mm. The mold containing 60 g sandy soil in the presence of Na-MMT (0.0%, 0.05%, 0.1%, 0.15%, and 0.2%) was submerged in the TCS and reacted by using a gaseous diffusion system. The experimental setup is shown in Figure S5. The TCS (4 L) was composed of bacteria (OD_600_ = 1.0) and the reaction solution (0.5 mol/L). All experiments were performed in triplicate.

### 2.4. Analytical Methods

The composition of the CaCO_3_ precipitates and Na-MMT were characterized by using XRD (Brook Company, Karlsruhe, Germany). The scan rate of 0.15 s step^−1^ was used and the range of 2θ was from 10° to 60°. 

A total of 0.5 mg of the samples and 200 mg of potassium bromide powder were taken and ground in a mortar. The pressure glass pieces machine was used to prepare glass pieces of the finely ground powders. The glass pieces of the samples were obtained with the pressure 10 T cm^−2^, and then the samples were characterized using Fourier-transform infrared spectroscopy (FTIR, SHIMADZU, Kyoto, Japan). The data were obtained over the wavenumber range of 400–4000 cm^−1^.

The morphologies of the CaCO_3_ precipitates and Na-MMT were observed using SEM (Zeiss Supra 55, Karlsruhe, Germany). Scanning was performed using an accelerating voltage of 25 kV. The PHS-3C PH meter and a calcium ion electrode were used to measure pH and the concentrations of the Ca^2+^ ions. Bacterial concentrations were measured using a UV spectrophotometer (Model, 752, Shimadzu Company, Kyoto, Japan company). 

## 3. Results 

### 3.1. Mechanism of Na-MMT on CaCO_3_ Precipitates

**Figure 1:**
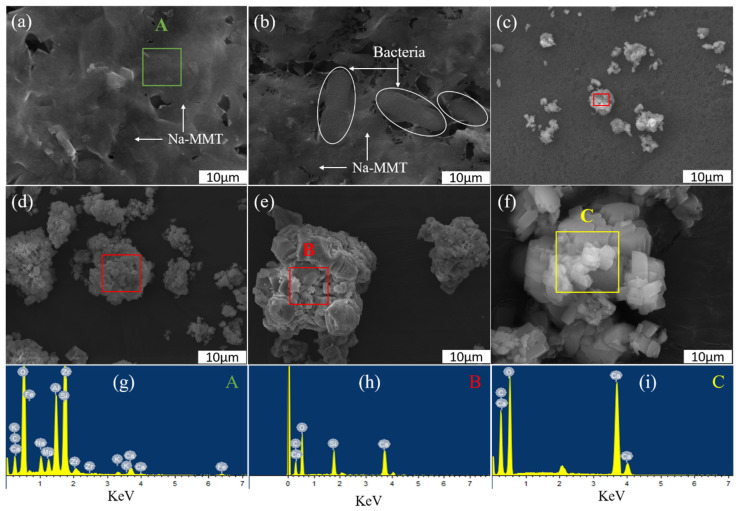
The SEM images of NA-MM (**a**) and CaCO_3_ precipitates with Na-MMT obtained at various times: 1 min (**b**), 30 min (**c**), 60 min (**d**), 2 h (**e**), 24 h (**f**), respectively. The EDS patterns of Na-MMT and CaCO_3_ precipitates from the marked area A (**g**), B (**h**) and C (**i**).

The mechanism of the MICP process incorporated with Na-MMT is presented in Figure 1. The morphology of Na-MMT is shown in Figure 1a. The EDS result (Figure 1g and Table S2) proved the element composition of Na-MMT. With the addition of Na-MMT in TCS, the bacteria were adsorbed onto the Na-MMT surface, as shown in Figure 1b. After 30 min, CaCO_3_ crystals and aggregates of CaCO_3_ crystals were observed, as shown in Figure 1c. In 2 h, the aggregate of CaCO_3_ crystals became larger with the reaction progress, as shown in Figure 1e. The EDS data (Figure 1h and Table S2) show that the aggregate of the CaCO_3_ crystal consisted of CaCO_3_ and Na-MMT. By 24 h, the shape of precipitates became more regular. Meanwhile, the only rhombohedral CaCO_3_ crystals were found; Na-MMT was not found, as shown in Figure 1i and Table S2, which indicated that Na-MMT was encapsulated in the CaCO_3_ crystals, serving as nucleation sites. The above results indicate that the bacteria can be adsorbed onto the Na-MMT surface. As reported in the previous literature, Ca^2+^ ions can be adsorbed by negatively charged bacteria [12,37,38], and intercalate into Na-MMT [39]. Afterwards, ${\mathrm{CO}}_{3}^{2-}$, formed via the hydrolysis of urea catalyzed bacterial urease, reacted directly with Ca^2+^ cross-linked with Na-MMT in the presence of bacteria to form CaCO_3_ crystals. Therefore, the growth of CaCO_3_ crystals can be achieved by the MICP process combined with Na-MMT. 

### 3.2. Influence of Na-MMT on CaCO_3_ Precipitates

**Figure 2:**
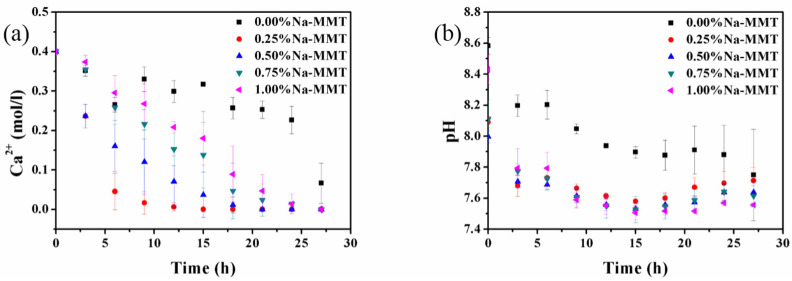
The change in the concentration of the Ca^2+^ ions (**a**) and pH (**b**) with Na-MMT concentrations.

The amount of CaCO_3_ precipitates was influenced by Na-MMT. Thus, the concentrations of Na-MMT were crucially important in the MICP process. A change trend of Ca^2+^ concentration under the effect of Na-MMT concentrations was revealed (Figure 2a). The result showed that the introduction of Na-MMT proved to be effective in accelerating the reaction of the Ca^2+^ ions compared to the reaction without Na-MMT. When the concentration of Na-MMT was 0.25%, 0.5%, 0.75%, and 1%, the changes in the trend of the Ca^2+^ concentration were the same. However, the fastest reaction rate of Ca^2+^ was observed with 0.25% Na-MMT. Finally, the Ca^2+^ was completely reacted in the presence of 0.25% Na-MMT, and this occurred at least 15 h earlier than when Na-MMT was absent. The changes in pH, with and without Na-MMT, are shown in Figure 2b. In the beginning, the pH was lower with the presence of Na-MMT. The reason for the decrease in pH was that the polysaccharide produced by bacteria consisted of alginic acid and gellan gum. Gellan gum is a high-molecular tetrasaccharide containing one acidic residue in repeating units [18,40]. Afterwards, the polysaccharide was not produced with bacteria as nuclear sites. Nevertheless, the pH drastically decreased between 0 h and 12 h in the presence of Na-MMT. This was because that the concentration of carbonate decreased, and the acid dissociation equilibrium changed as the carbonate precipitated. By the Le Chatelier principle, the decrease in the carbonate in the solution (by the precipitation) shifted the equilibrium direction towards the production of more carbonate (i.e., the dissociation of carbonic acid and bicarbonate). Hence, more H^+^ was produced and the pH decreased.

**Figure 3:**
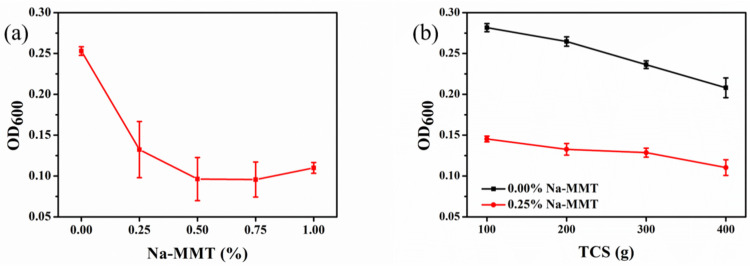
OD_600_ of bacteria with various Na-MMT concentrations (**a**) and TCS quantities in the absence of and in the presence of 0.25% Na-MMT (**b**).

The bacterial adsorption capacity in the presence of Na-MMT concentrations is shown in Figure 3a. The adsorption capacity of bacteria was the same with various concentrations of Na-MMT but the adsorption capacity was the best with 0.5% Na-MMT. Compared with the control sample, the adsorption capacity of bacteria was improved at most by 62% in the presence of 0.5% Na-MMT. The bacterial adsorption capacity of TCS of different quantities without and with 0.25% Na-MMT is shown in Figure 3b. Whether Na-MMT was added or not, the bacterial adsorption capacity increased when TCS increase. The reason was that the Ca^2+^ ions could be adsorbed by the negatively charged bacteria, which led to precipitates of bacteria. Therefore, the OD_600_ values of bacteria decreased significantly with the increase in the number of Ca^2+^ ions. Nevertheless, compared with the absence of Na-MMT, more bacteria were adsorbed in the presence of Na-MMT.

**Figure 4:**
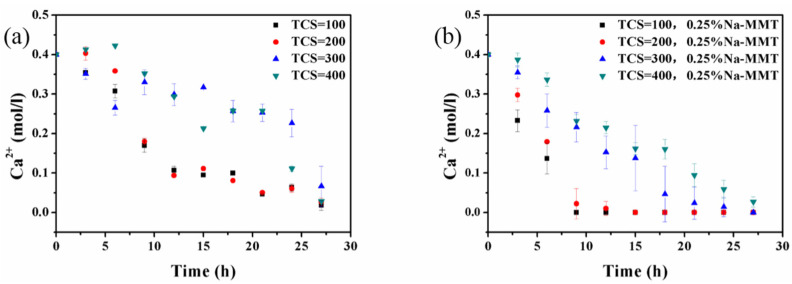
The change in the concentration of Ca^2+^ ions with different quantities of TCS without Na-MMT (**a**) and with 0.25% Na-MMT (**b**).

The reaction of Ca^2+^ ions decreased with increments of TCS in the absence of Na-MMT (Figure 4a) and the presence of Na-MMT (Figure 4b). The development of Ca^2+^ ion concentrations was the same in various TCS quantities. The results shown in Figure 4b are consistent with those shown in Figure 4a. However, compared with the traditional MICP method (Figure 4a), the reaction of Ca^2+^ ions was significantly enhanced in the presence of Na-MMT (Figure 4b).

**Figure 5:**
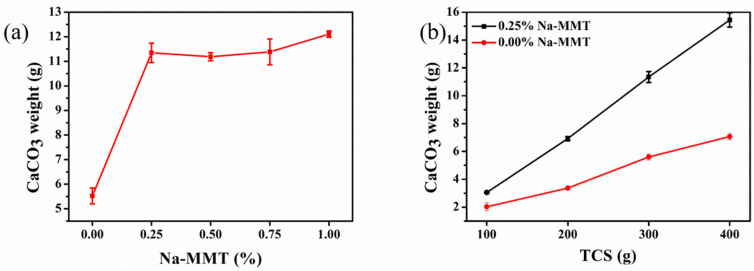
The total mass of CaCO_3_ precipitates with different Na-MMT concentrations (**a**) and with TCS quantities with and without Na-MMT (**b**).

The amount of CaCO_3_ precipitates in the presence of Na-MMT increased by about 118% compared to the traditional MICP method (Figure 5a). The amount of CaCO_3_ precipitates catalyzed by the use of urease of bacteria under the effect of Na-MMT concentrations was the same. This was because the urea completely decomposed and the Ca^2+^ ions completely reacted to form the CaCO_3_ precipitates when Na-MMT was added. Figure 5b shows that the precipitates of CaCO_3_ increased with an increase in TCS whether Na-MMT was added or not. Meanwhile, the presence of Na-MMT enhanced the amount of CaCO_3_ precipitates in the MICP process. Compared to the traditional MICP method, the amount of CaCO_3_ precipitates was significantly higher under the effect of Na-MMT. Theoretically, the precipitate of CaCO_3_ should have been 12 g in the 300 g TCS. However, the actual precipitate of CaCO_3_ was 6 g in the 300 g TCS, as shown in Figure 5a. Therefore, we thought that increasing the amount of TCS was not conducive to the precipitates of CaCO_3_. The actual precipitate of CaCO_3_ was 12 g in the 300 g TCS with the introduction of Na-MMT (Figure 5a), which confirmed that the actual precipitates of CaCO_3_ in the presence of Na-MMT reached the theoretical value.

### 3.3. Influence of Na-MMT on the Polymorph and Morphology of CaCO_3_ Precipitates

**Figure 6:**
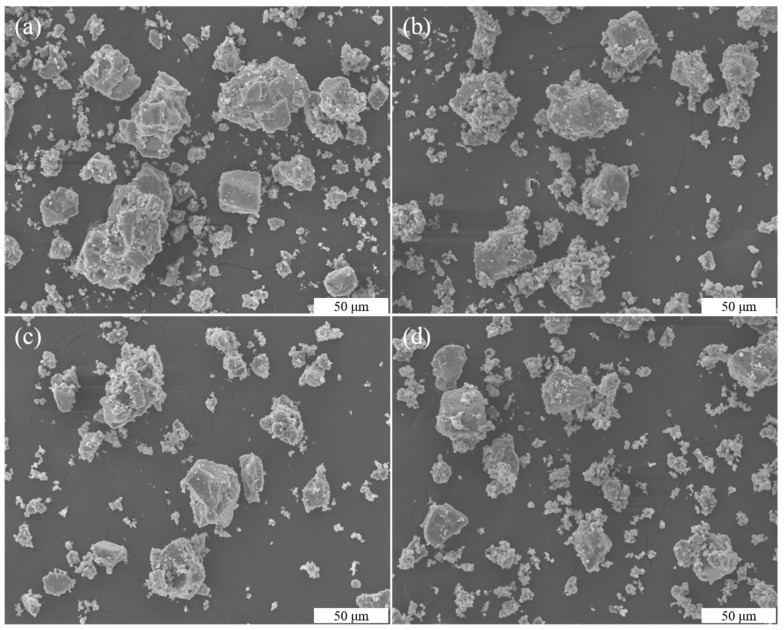
SEM images of CaCO_3_ precipitates with different quantities of TCS (TCS = 100 (**a**), 200 (**b**), 300 (**c**), and 400 g (**d**)).

The morphology of the CaCO_3_ precipitates obtained with different quantities of TCS in the absence of Na-MMT was investigated by using SEM (Figure 6a–d). Irregular and regular rhombohedral CaCO_3_ precipitates were obtained with different quantities of TCS. The reason for the irregularity of the precipitates was that the concentration of TCS was too high. At the same time, the particle size distribution was the same under different quantities of TCS.

**Figure 7:**
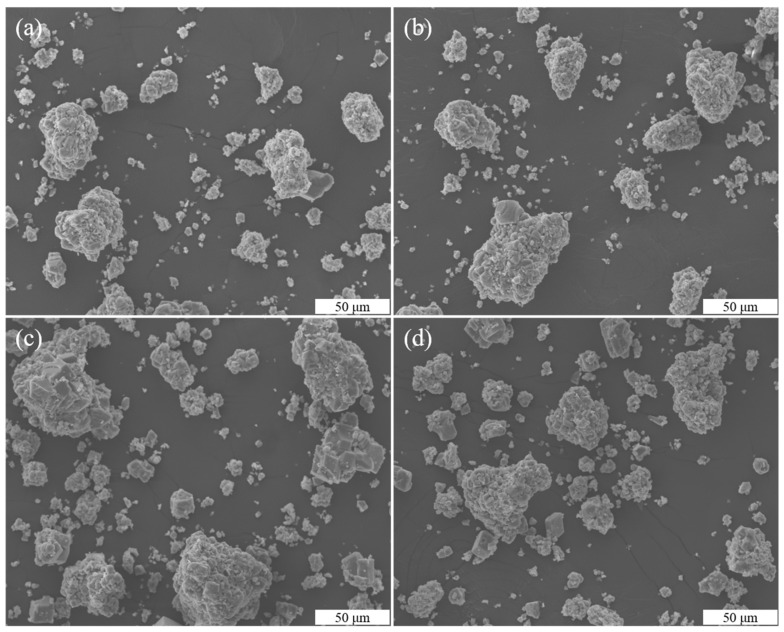
SEM images of CaCO_3_ crystals with different quantities of TCS with 0.25% Na-MMT (TCS = 100 (**a**), 200 (**b**), 300 (**c**), and 300 g (**d**)).

The morphologies of the CaCO_3_ precipitates that were prepared with different quantities of TCS with 0.25% Na-MMT were also investigated using SEM (Figure 7a–d) and were compared to the CaCO_3_ precipitates in the absence of Na-MMT. The agglomerated CaCO_3_ crystals were observed in the presence of Na-MMT. Meanwhile, the individual rhombohedral precipitates were rarely found. The above results indicate that CaCO_3_ crystal particles could be agglomerated by using Na-MMT during the MICP process, which is important because the agglomeration of the CaCO_3_ crystals may affect bio-cementation [29].

**Figure 8:**
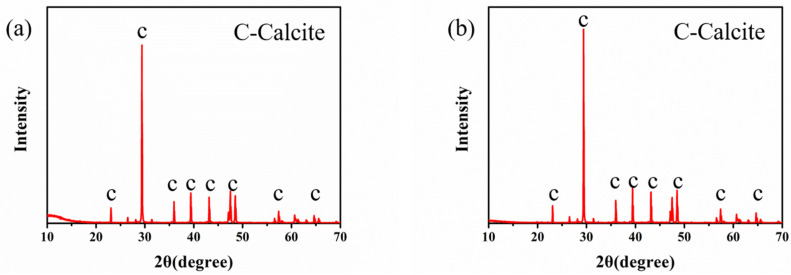
XRD patterns of CaCO_3_ precipitates catalyzed by bacteria (TCS = 100) without (**a**) and with 0.25% Na-MMT (**b**).

XRD analysis confirmed that with or without Na-MMT, the main component of CaCO_3_ precipitates was calcite at 27 h (Figure 8). This result is in line with that in Figure 1.

### 3.4. Influence of Na-MMT on Hydraulic Conductivity

**Figure 9:**
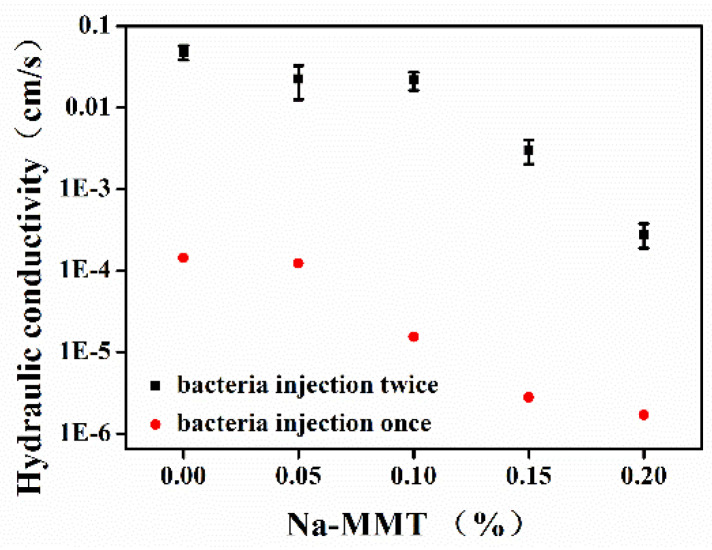
The hydraulic conductivity of consolidated sandy soil with different Na-MMT concentrations.

The hydraulic conductivity (HC) is shown in Figure 9. The use of Na-MMT did not decrease HC without the MICP process (The data are not displayed here). HC reduced with an increase in the concentrations of Na-MMT in the MICP process. The HC of the initial conditions of the consolidated sandy soil obtained by the MICP process in the absence of Na-MMT was 3.8 × 10^−2^ cm/s. When the concentrations of Na-MMT was 0.15%, the HC was decreased to 2.03 × 10^−3^ cm/s. The HC decreased to the range of 1.34 × 10^−3^ to 1.8 × 10^−6^ cm/s when TCS was injected twice. The HC of the sandy soil consolidated by MICP decreased to 1.8 × 10^−6^ cm/s in the presence of Na-MMT, which was the lowest HC that was able to be measured in the batch experiments.

**Figure 10:**
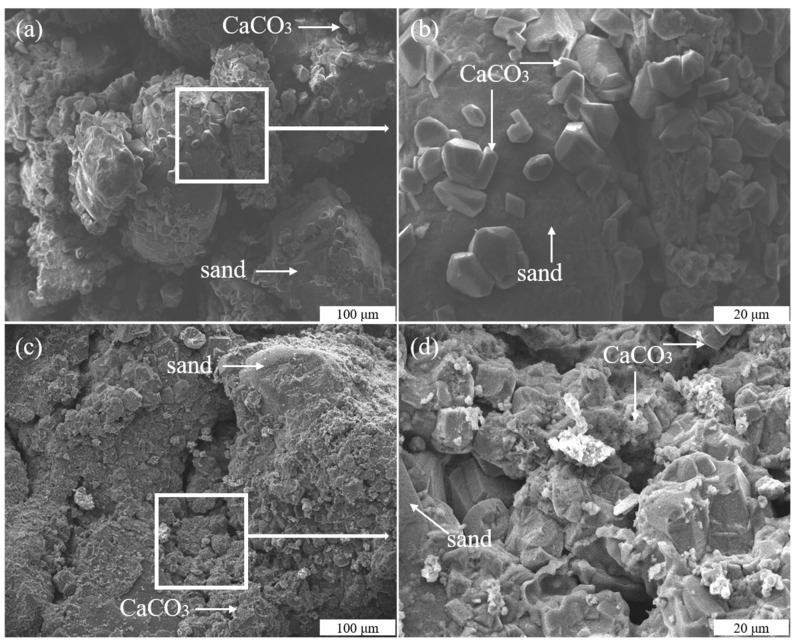
SEM images of the consolidated sandy soil samples without (**a**,**b**) and with NA-MMT (**c**,**d**).

The images of SMEs show that cementation mainly occurred at the contact points between the sandy soil particles (Figure 10). Meanwhile, a small amount of CaCO_3_ was adsorbed on the surface of sandy soil particles (Figure 10a). The reason for this was the low adsorption capacity of the bacteria on the surface of the sandy soil [41]. Meanwhile, the large gap between the sandy soil particles was not filled tightly, which may have been due to the low output of CaCO_3_ between the sandy soil gap (Figure 10b). Thus, good hydraulic conductivity was achieved. The SEM images of the consolidated sandy soil by the MICP process with Na-MMT are shown in Figure 10c,d. These images show that CaCO_3_ precipitates tightly filled the voids between the sandy soil particles in the presence of Na-MMT. Na-MMT was not only a carrier for the formation of the CaCO_3_ precipitates, but also enhanced the yield of CaCO_3_ precipitates at the contact points. That is why there was a denser structure between the sandy soil particles during the MICP process in the presence of Na-MMT. Thus, a lower hydraulic conductivity can be achieved because of the more pronounced bio-clogging and bio-cemented effect. 

## 4. Discussion

The effect of Na-MMT on MICP has been evaluated by using *Sporosarcina pasteurii*. Na-MMT is pure and natural and is not pathogenic for human beings, plants, or animals. Thus, the application of Na-MMT will not lead to a foreseeable issue. Na-MMT in various concentrations were used for the batch experiments. The plot showed that the optimum concentration of Na-MMT was 0.25% to achieve the maximum reaction rate of the Ca^2+^ ions. The above result is important for altering the reaction time of the MICP process, which has great value for field application [42,43,44].

Generally, a highly specific surface and the presence of Na^+^ and Ca^2+^ of Na-MMT may contribute to bacterial sorption. Ca^2+^ ions can be adsorbed by Na-MMT [45], which led to an increase in the positive charge around Na-MMT. Therefore, more negatively charged bacteria were immobilized by cations of Na-MMT in a rich calcium environment [46,47]. The strong adsorption capacity of Na-MMT is also an important reason [48,49]. Meanwhile, the literature has also reported that the microbial activity (such as a faster reaction rate), the distribution, and the biomineralization of bacteria could be altered by the sorption of MMT [50]. Thus, an understanding of the sorption of bacteria on the Na-MMT surface is of great geological significance.

The amount of the CaCO_3_ precipitates was greatest in TCS with 0.25% Na-MMT because Ca^2+^ was almost completely deposited. The reason was that Na-MMT was added, the urea was completely decomposed, and the Ca^2+^ ions completely reacted to form the CaCO_3_ precipitates. In theory, 0.4 mol of urea can be decomposed to form a precipitate of 0.4 mol of CaCO_3_ in the MICP process. Nevertheless, the above result was not found with different quantities of TCS without Na-MMT. This was because the activity of urease determines the hydrolysis rate of urea rather than the content of minerals in the reaction medium [51]. At the same time, the activity of bacteria may be inhibited due to excessive Ca^2+^ concentrations, and consequently may have affected the production of CaCO_3_ [52]. However, the precipitates of CaCO_3_ reached the theoretical value in the presence of Na-MMT. The weight of CaCO_3_ generated by the MICP process was proportional to the TSC of consolidated sandy soil [53,54], and inversely proportional to the hydraulic conductivity of consolidated sandy soil samples. Therefore, the production of CaCO_3_ was very crucial.

The properties of the CaCO_3_ precipitates, including their distribution, particle size, morphology, and specific surface area, have a tremendous impact on their application in industries [55]. In this study, compared to the traditional MICP method, the agglomeration of CaCO_3_ crystals occurred due to the fact that Na-MMT has a highly specific surface and a strong adsorption capacity. XRD analysis confirmed that in the presence of and in the absence of Na-MMT, the main component of the precipitates obtained was calcite, as shown in Figure 6. The basic components of Na-MMT were not detected. Therefore, we thought that Na-MMT can be used as nucleus sites. 

## 5. Conclusions

The addition of Na-MMT has great potential to improve the efficiency of the MICP process. The results revealed that Na-MMT accelerated the reaction of Ca^2+^ ions, increased the amount of CaCO_3_ precipitates and bacteria adsorbed, and reduced the hydraulic conductivity of consolidated sandy soil samples. The characterization of SEM and XRD confirmed that individual CaCO_3_ precipitates were observed in the absence of Na-MMT and that the agglomeration of the precipitates occurred in the presence of Na-MMT. Calcites were obtained with and without Na-MMT. According to the above experiment, adding Na-MMT is an effective way to enhance the effect of the MICP process.

## Data Availability

We choose to exclude this statement because the study did not report any data.

## Supplementary Materials

The following are available online: Figure S1: Grading size distribution curves of NA-MMT (A) and the sandy soil (B) used in sample preparation, Figure S2: SEM images of NA-MMT (A) and the sandy soil (B) used in sample preparation, Figure S3: Characterization of NA-MMT investigated using XRD (A) and FTIR (B), Figure S4: Characterization of NA-MMT investigated using BET, Figure S5: Schematic drawing of the batch reactor, Table S1: IR Bands for NA-MMT Used in FTIR, Table S2: The EDS data of NA-MMT and CaCO3.

## Sample Availability

Samples of the compounds are available from the authors.
