# Supplementary material for: Role of Na-Montmorillonite on Microbially Induced Calcium Carbonate Precipitation
Source: Molecules. 2021 Oct 14;26(20):6211. doi: 10.3390/molecules26206211 (PMC8538570; doi:10.3390/molecules26206211)
Supplement: Supplementary file 1 [file molecules-26-06211-s001.zip › molecules-1368551-supplementary.pdf]

Role of Na-montmorillonite on Microbially Induced Calcium Carbonate Precipitation

Guowang Tang [1], Cangqin Jia[1,2*], Guihe Wang [1], Peizhi Yu[1], Haonan Zhang[1]

[1]School of Engineering and Technology, China University of Geosciences, Beijing 100083, P R of China.

[2]Key Laboratory of Deep Geodrilling Technology, Ministry of Land and Resources, China University of Geosciences, Beijing 100083, P R of China.

*Corresponding author, Cangqin Jia[1,2] e-mail: jiacangqin@cugb.edu.cn. Guowang Tang[1] e-mail: 3002180015@cugb.edu.cn; Guihe Wang[1] wanggh@cugb.edu.cn; Peizhi Yu e-mail: 2011010003@cugb.edu.cn; Haonan Zhang[1] e-mail: 3002190015@cugb.edu.cn

There are 4 pages in supporting information, include 5 figures and 2 table.

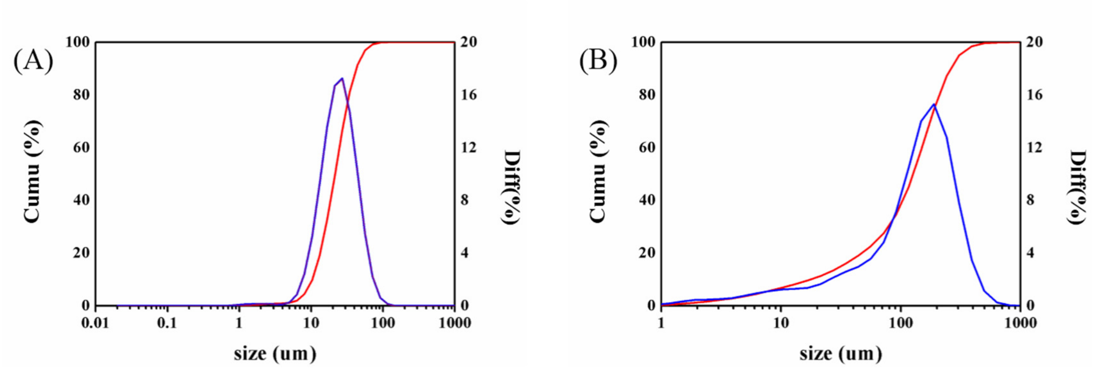

Figure S1. Grading size distribution curves of NA-MMT (A) and the sandy soil (B) used in sample preparation.

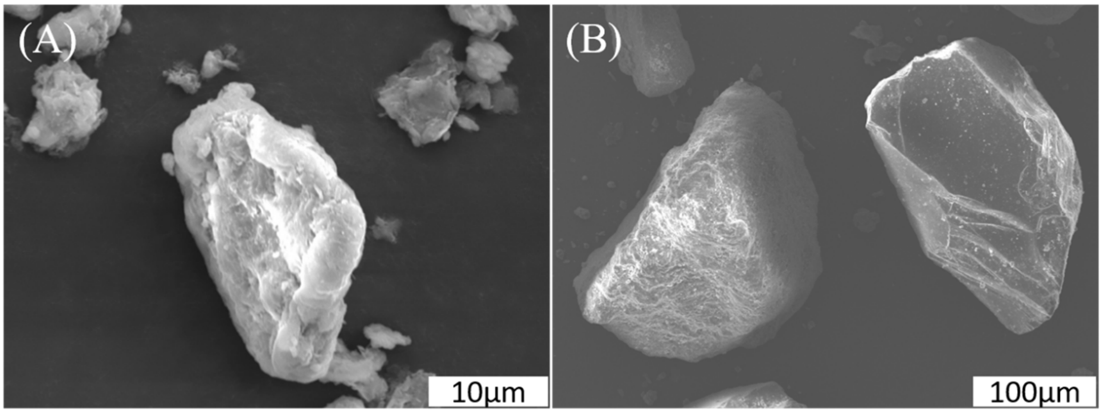

Figure S2. SEM images of NA-MMT (A) and the sandy soil (B) used in sample preparation.

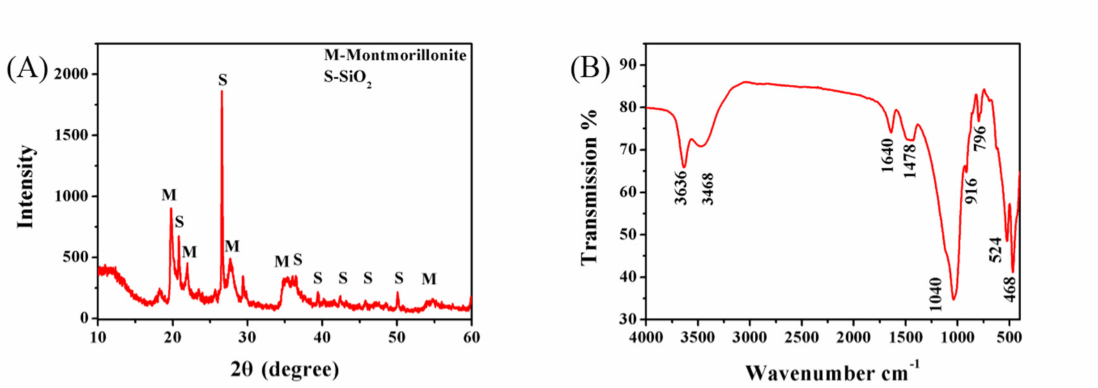

Figure S3. Characterization of NA-MMT investigated using XRD (A) and FTIR (B).

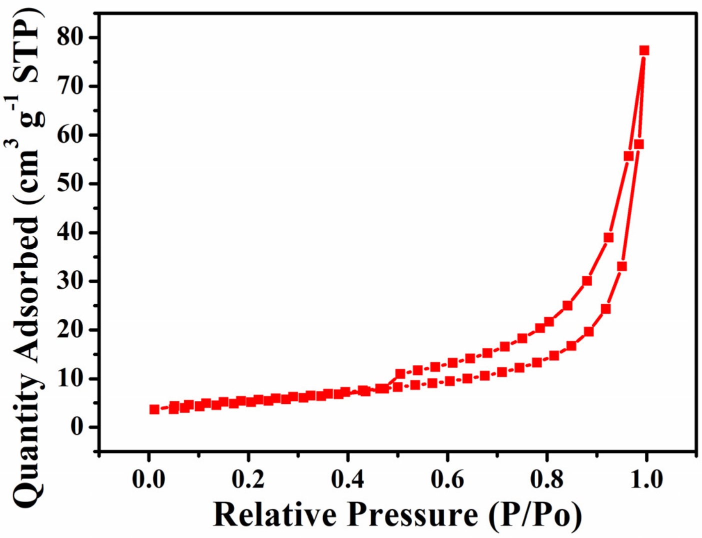

Figure S4. Characterization of NA-MMT investigated using BET

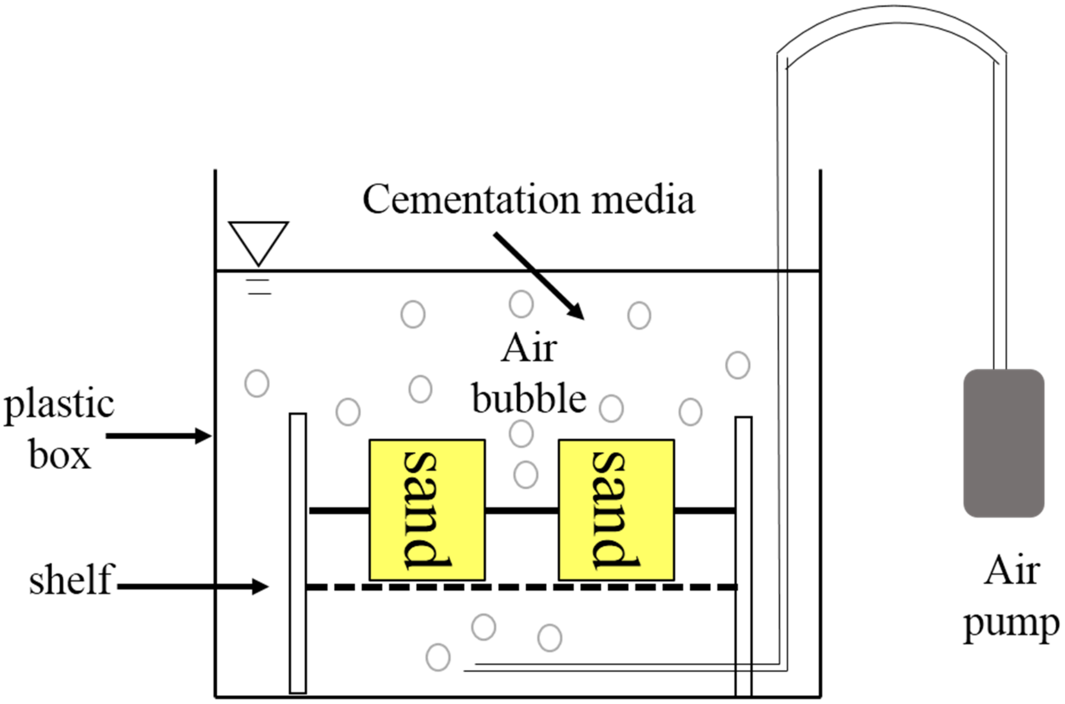

Figure S5. Schematic drawing of the batch reactor.

Table S1. IR Bands for NA-MMT Used in FTIR.

band (cm^{-1})	Transmittance (%)	Assignments
3636	65.9	Al—O–H
3468	70.8	H–O–H stretch
1640	74.1	H–O–H str.
1040	34.7	Si-O
916	64.7	Al—O–H str
796	76.8	Si–O str., Si–O–Al
524	48.5	Si–O str., Si–O–Al
468	41.2	Si–O，Si–O–Al

Table S2. The EDS data of NA-MMT and $CaCO_3$.

Element	A		B		C	
	Weight (%)	Atomic (%)	Weight (%)	Atomic (%)	Weight (%)	Atomic (%)
C	12.08	18.14	17.92	26.29	20.86	30.43
O	54.21	61.09	56.21	61.91	53.17	58.22
Na	1.86	1.46				
Mg	1.33	0.98				
Al	7.12	4.76				
Si	18.57	11.92	2.29	1.44		
K	0.47	0.22				
Ca	1.72	0.77	23.58	10.37	25.97	11.35
Fe	1.07	0.34				
Zr	1.58	0.31				
